# Comparative transcriptomic analysis of reproductive characteristics of reciprocal hybrid lineages derived from hybridization between *Megalobrama amblycephala* and *Culter alburnus*

**DOI:** 10.1186/s12863-023-01141-6

**Published:** 2023-08-12

**Authors:** Xue Ding, Yifei Zhang, Die Li, Jia Xu, Chang Wu, Xiaojuan Cui, Yuandong Sun

**Affiliations:** 1 School of Life Science and Health, Hunan University of Science and Technology, Xiangtan, 411201 Hunan China; 2 State Key Laboratory of Developmental Biology of Freshwater Fishes, Hunan Normal University, Changsha, 410081 Hunan China

**Keywords:** Distant hybridization, Hybrid lineages, Fertility, Reproductive characteristics, Transcriptome

## Abstract

**Background:**

Distant hybridization is an important breeding technique for creating new strains with superior traits by integrating two different genomes. Successful hybridization of *Megalobrama amblycephala* (Blunt snout bream, BSB, 2n = 48) and *Culter alburnus* (Topmouth culter, TC, 2n = 48) was achieved to establish hybrid lineages (BT and TB), which provide valuable materials for exploring the mechanisms of distant hybridization fertility. In this study, the gonadal tissue transcriptomes of BSB, TC, BT-F_1_, and TB-F_1_ were sequenced using Illumina high-throughput sequencing technology to analyze the reproductive characteristics of BT and TB.

**Results:**

Differential gene expression analysis showed that the differentially expressed genes in BT *vs* BSB and BT *vs* TC were mainly enriched in signaling pathways not directly associated with meiosis. While, the differentially expressed genes of TB *vs* BSB and TB *vs* TC were mainly enriched in pathways related to meiosis, and most of them were down-regulated, indicating that meiosis is suppressed in TB. Under-dominance (UD) genes were enriched in pathways related to meiosis and DNA repair in TB. Over-dominance (OD) genes were enriched in MAPK signaling pathway, expression level dominance-BSB (ELD-B) genes were enriched in pathways related to steroid hormone synthesis and expression level dominance-TC (ELD-T) genes were not significantly enriched in any pathway in both BT and TB.

**Conclusions:**

These results suggest that meiotic progression may not be affected in BT, whereas it is clearly inhibited in TB. Offspring of *M*. *amblycephala* maternal parent may have better genomic compatibility and fertility. Our study provides important information on the molecular mechanisms of breaking reproductive isolation in distantly hybridized fertile lineages.

**Supplementary Information:**

The online version contains supplementary material available at 10.1186/s12863-023-01141-6.

## Background

Distant hybridization refers to the hybridization between two species that are genetically distant, ranging from different species to different genera or even further away. This process enables the combination of genomes from two different species, leading to increased genetic variation, and the creation of new varieties with desirable traits [1, 2]. Distant hybridization has become an important breeding method extensively used in plants and animals. For example, distant hybrid crops such as corn (*Zea mays*) [3], and Pepper (*Capsicum annuum* L.) [4] have been widely cultivated in China. In fish, successful distant hybridization cases, such as *Carassius auratus* red var. (♀) × *M. amblycephala* (♂) [5] and *M. amblycephala* (♀) × *Xenocypris davidi* (♂) [6] have also been reported.

*M. amblycephala* and *C. alburnus* are freshwater fishes belonging to the order Carpiformes, Cyprinidae, Culterinae, the former in *Megalorama* and the latter in *Culter,* both are economically important cultured freshwater fishes in China [7, 8]. *M. amblycephala* is an herbivorous fish with strong disease resistance, fast growth rate, and high survival rate [9]. *C. alburnus,* on the other hand, has the advantages of fresh and tender meat, strong disease resistance, and stress resistance [10]. Professor Shaojun Liu and his team from Hunan Normal University have successfully established allodiploid BT hybrid lineage (♀*M. amblycephala* × ♂*C. alburnus*, 2n = 48) and TB hybrid lineage (♀*C. alburnus* × ♂*M. amblycephala*, 2n = 48) through intergeneric intercross between *M. amblycephala* and *C. alburnus*, overcoming the reproductive barriers. The BT and TB lineages are fertile in both sexes, with normal gonadal development, and have been stably bred to the fifth (BT-F_1-5_) and third (TB-F_1-3_) generations, respectively [11]. BT and TB hybrids are intermediate in shape between the two parents [12], and exhibit many physiological advantages, such as faster growth, better tolerance to hypoxia, and greater disease resistance [12, 13]. BT and TB have two sub-genomes from the parents and chimeric genes derived from the DNA of both parents [11]. They are herbivorous [13, 14]and have tender meat with significantly higher meat content than their parents [15–17]. At present, most studies focus on the molecular mechanisms of hybrid infertility [18], while few reports exist on hybrid fertility. Therefore, this study preliminarily explores the reproductive characteristics of BT-F_1_ and TB-F_1_ hybrid fish that have passed the reproductive barrier.

Hybrids from distant hybridization are typically infertile due to reproductive isolation making it necessary to overcome this barrier to establish fertile hybrid lineages. Reproductive isolation includes both pre-fertilization and post-fertilization reproductive barriers, with hybrid sterility falling under the latter category. Meiosis, an essential process for gametogenesis in sexual reproduction and a prerequisite for species stability, holds significant biological importance [19]. Current studies indicate that hybrid infertility is primarily caused by disorder in heterochromosome pairing and segregation during meiosis [20]. Examples of hybrids with hybrid sterility include the horse and donkey hybrid [21], allotriploid crucian carp [22], and sterile male hybrids of *Cobitis taenia* and *C. elongatoides* [23]. In addition, studies have demonstrated that aberrant expression of genes involved in cell cycles [24], reproduction [25–32], apoptosis [26, 33], and abnormal hormone levels [27] may contribute to sterility of hybrid fish.

There are few reports on fertile lineages resulting from distant hybridization, but the creation of fertile hybrid lineages of BT and TB provides a valuable resource for investigating methods to overcome reproductive isolation. In this study, we used transcriptome sequencing technology to analyze the expression of meiosis-related genes between the hybrid and the original parents in BT-F_1_ and TB-F_1_ to identify the key genes and regulatory pathways. This research sheds light on the molecular mechanisms behind overcoming reproductive barriers in hybrid fish and is of great importance for creating fish germplasm resources.

## Materials and methods

### Fish and sampling

The experimental fish used in this study were provided by the State Key Laboratory of Developmental Biology of Freshwater Fishes, Hunan Normal University, China. For each of the 1-year-old BSB, TC, BT-F_1_ and BT-F_1_, nine fish were collected from the same batch and raised under the same conditions. All experiments were approved by the Animal Care Committee of Hunan Normal University. The fish were anesthetized with 100 mg/L MS-222 (Sigma-Aldrich, St Louis, MO, USA) prior to dissection. The gonads were then dissected, immediately treated with liquid nitrogen and stored at -80 ℃. Nine fish were collected from each group, and three fish were randomly assigned to each of three biological replicates.

### Extraction of total gonadal RNA

Nine fish were collected from each of the four groups: BSB, TC, BT-F_1_, and TB-F_1_. The fish from each group were divided into three independent biological replicates, resulting in a total of 12 gonadal tissue samples, labeled as BSBG_1-3, TCG_1-3, BTG_1-3, and TBG_1-3. Total RNA was extracted from each sample using Trizol (Invitrogen, USA). To extract RNA, gonadal tissue (60 mg) was homogenized in a 1.5 mL EP tube containing 1 mL of Trizol. After homogenization, the samples were centrifuged at 12,000 rpm for 10 min at 4 °C, and the supernatant was transferred to another clean EP tube. Next, 200 μL of trichloromethane was added to the tube, which was then shaken vigorously for 15 s, left at room temperature for 2–3 min, and centrifuged again at 12,000 rpm for 10 min at 4 °C. After centrifugation, the upper aqueous phase was transferred into another EP tube, to which 500 μL isopropanol was added. The liquid was thoroughly mixed, left at room temperature for 10 min, and then centrifuged at 12,000 rpm for 10 min at 4 °C. The supernatant was discarded, and the RNA precipitation was washed with 1 mL of 75% ethanol, vortexed, shaken, and centrifuged at 7500 rpm for 5 min at 4 °C to remove residual ethanol. Subsequently, the RNA precipitation was air-dried in a biosafety cabinet for 5 min and finally dissolved with 30 μL DEPC water for 10 min. The extracted total RNA was examined using 1.5% agarose gel electrophoresis, a NanoPhotometer spectrophotometer and an Aglient 2100 (Termo Fisher Scientifc, USA).

### RNA sequencing

Total RNA was purified using Oligo (dT) magnetic beads, and the obtained mRNA was randomly interrupted with divalent cations in Fragmentation Buffer. The mRNA fragment was used as a template for reverse transcription to synthesize the first strand cDNA, followed by the addition of dNTPs to synthesize the second strand cDNA using DNA polymerase I. After purification, the double-stranded cDNA was subjected to end repair, addition of an A-tail, ligation of sequencing adapters and length screening (about 370–420 bp), followed by PCR amplification and re-purification of PCR products to construct the final cDNA library. After the library construction, the Agilent 2100 was used to detect the size of the inserted fragment, and QPCR technology was employed to accurately quantify the effective concentration of the library (effective concentration > 2 nM) to ensure the quality of the library. Illumina sequencing was performed after the sequencing library was qualified.

### Data analysis

The raw sequencing data were filtered by removing adapter, reads with low sequencing quality and reads containing N (undeterminable base), to obtain high-quality clean data to ensure the reliability of subsequent analysis. The clean reads were aligned to the reference genome (GCF_018812025.1_ASM1881202v1) using HISAT2 v2.0.5 [34] to obtain the positioning information of each read. The reads mapped to each gene were calculated using featureCounts (1.5.0-p3) [35], and FPKM values were calculated for each gene based on its length and the mapped reads [36]. FPKM normalizes for sequencing depth and gene length, and is a commonly used method for estimating gene expression levels. Differential gene expression analysis between the two comparative groups was performed using DESeq2 (1.20.0) [37]. The method of Benjamini and Hochberg was used to adjust the resulting P-values to control the false discovery rate (FDR). Genes with adjusted P-values ≤ 0.05 were considered differentially expressed. The screening criteria for differential genes were set as | log2 (FoldChange) |≥ 1 and padj ≤ 0.05. The clusterProfiler (3.8.1) software was used to perform GO functional enrichment analysis and KEGG pathway enrichment analysis of the differential gene sets. Significant enrichment was defined as padj < 0.05 for both GO and KEGG enrichment analyses. GO is a comprehensive database that describes the functions of genes and proteins, which can be classified into three parts: biological processes, cellular components, and molecular functions [38]. KEGG is a comprehensive database that provides genomic, chemical and systemic functional information [39].

### Differentially expressed gene classification

Following the method described in the article of Zhou Y et al. [40], differentially expressed genes were classified as additive or non-additive, and non-additive genes were further classified into four expression patterns, ELD-B (expression level dominance-BSB), indicating expression levels similar to BSB, ELD-T (expression level dominance-TC), indicating expression levels similar to TC, OD (over-dominance), indicating higher expression levels than parents; and UD (under-dominance), indicating lower expression levels than parents. The Novo clound platform was used to analyze these genes based on their classification. Only non-additive genes were analyzed in this study.

### qRT-PCR validation

To verify the accuracy of the transcriptome sequencing results, 20 genes related to biological events such as oocyte meiosis and homologous recombination were selected for qRT-PCR analysis. Total RNA was reversely transcribed into cDNA using PrimeScript™ RT reagent Kit with gDNA Eraser (Perfect Real Time) kit (Takara, Japan). qRT-PCR reactions were performed using the TB Green® *Premix Ex Taq™* II (Tli RNaseH Plus) kit (Takara, Japan). The PCR reaction system contained the following ingredients: 1 μL template cDNA, 10 μl TB Green *Premix Ex Taq* II (Tli RNaseH Plus)(2 ×), 1 μL upper/downstream primers, and 7 μL RNase Free dH2O. qRT-PCR was performed using the Bio-Rad CFX96 Real-Time System with a cycling program of 30 s at 95 °C; 40 cycles, each at 95 °C for 15 s, at 60 °C for 15 s; at 72 °C for 30 s, and finally at a rate of 0.5 °C every 5 s from 65 °C to 95 °C to generate a melting curve, demonstrating the absence of dimeric primers and generation of non-specific amplification. *Ef1-α* and *β-actin* were selected as reference genes. The qRT-PCR experiments were repeated three times for each gene. The relative expression levels of the 20 genes in different samples were calculated using the 2^−ΔΔCT^ method. The primer sequences used are listed in Additional file 1.

## Results

### Summary statistics of transcriptome sequencing data

**Fig. 1:**
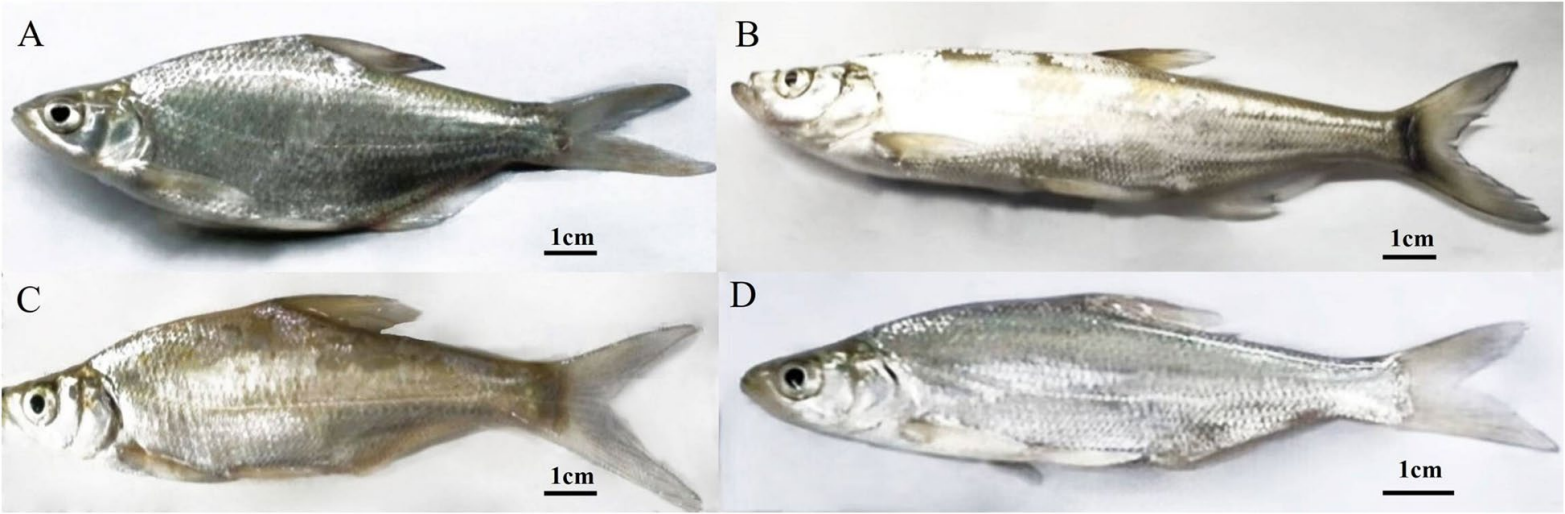
*M. amblycephala*, *C. alburnus* and their hybrids. **A** *M. amblycephala* (BSB), **B** *C. alburnus* (TC), **C** BT-F_1_, **D** TB-F_1_

To investigate the reproductive characteristics of BT and TB, the gonadal cDNA libraries of one-year-old BSB, TC, BT-F_1_ and TB-F_1_ were constructed respectively, and transcriptome sequencing analysis was performed using the Illumina sequencing platform (Fig. 1). The summary of quality control results of transcriptome sequencing data is shown in Table 1. The average number of clean reads obtained per sample was 7.31G, with an average Q20 of 97.15%, an average Q30 of 92.93%, a GC content ranging from 47.88% to 52.97%, and an average mapping genome ratio of 80.425 for the 12 samples.

**Table 1:** Summary of transcriptome sequencing data quality control

Sample ID	Raw reads	Raw bases	Clean reads	Clean bases	Q20 (%)	Q30 (%)	GC content (%)	Total Mapping Genome Ratio (%)
BSBG_1	53,615,366	8.04G	53,012,390	7.95G	97.7	93.98	48.75	91.8
BSBG_2	34,254,722	5.14G	33,830,450	5.07G	96.75	91.75	49.38	83.62
BSBG_3	30,522,678	4.58G	29,983,046	4.5G	94.79	88.85	52.97	75.97
TCG_1	80,702,816	12.11G	80,000,290	12.0G	97.74	94.13	48.99	75.76
TCG_2	67,579,204	10.14G	66,943,960	10.04G	97.6	93.84	48.95	76.08
TCG_3	50,988,020	7.65G	50,396,748	7.56G	97.67	93.88	48.61	78.58
BTG_1	46,785,306	7.02G	46,224,544	6.93G	97.57	93.66	48.6	83.76
BTG_2	41,872,340	6.28G	41,073,866	6.16G	97.55	93.77	49.09	79.48
BTG_3	40,782,188	6.12G	40,150,030	6.02G	97.58	93.68	47.88	81.64
TBG_1	25,774,252	3.87G	25,242,040	3.79G	95.81	89.93	50.74	75.86
TBG_2	66,684,520	10G	65,465,158	9.82G	97.17	93.16	48.76	81.88
TBG_3	53,179,342	7.98G	52,374,882	7.86G	97.9	94.48	51.08	80.67

### Screening and functional analysis of differentially expressed genes

**Fig. 2:**
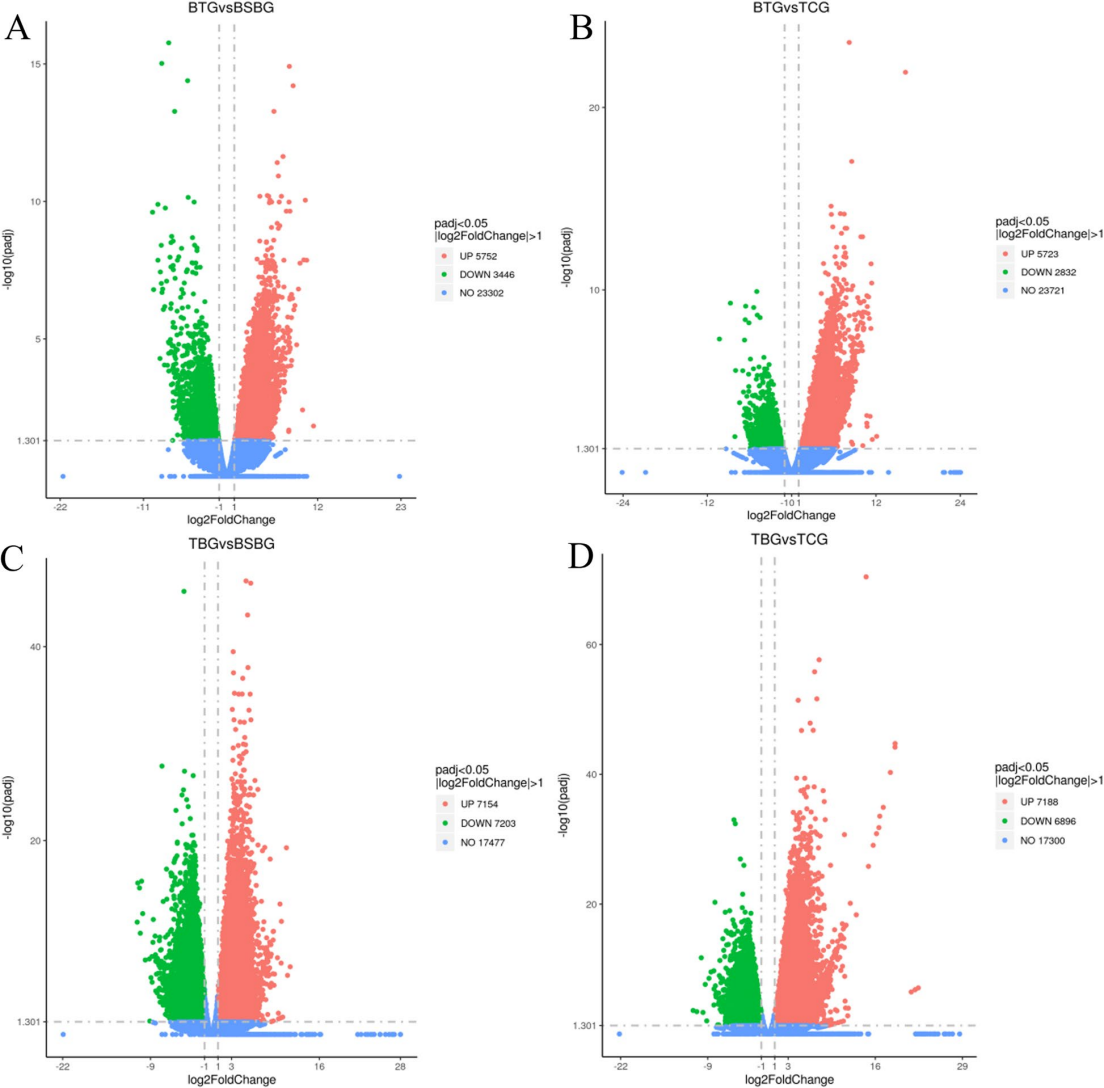
Volcano plot of differentially expressed genes in the four groups. **A **BT *vs* BSB, **B **BT *vs* TC, **C **TB *vs* BSB, **D **TB *vs* TC. The abscissa shows the log2FoldChange value, the ordinate is log10padj, and the blue dashed line indicates the threshold line for the differential gene screening criteria

**Fig. 3:**
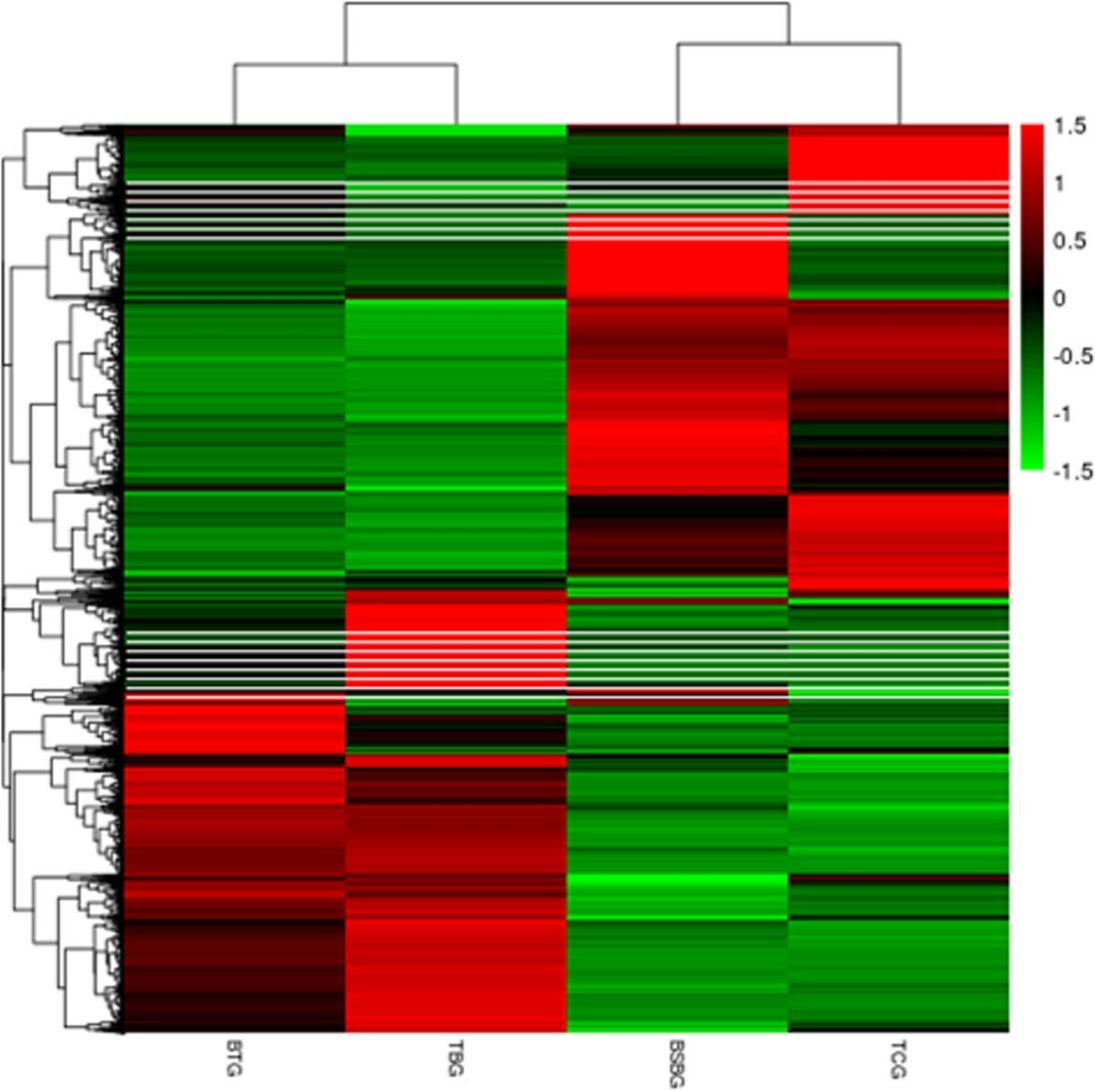
Heatmap showing the expression levels of differentially expressed genes. The horizontal axis represents the sample group names, and the vertical axis shows the normalized values of differentially genes FPKM. Red color indicates higher expression levels, and green color indicates lower expression levels

A total of 9198 differentially expressed genes (DEGs) were identified in BT *vs* BSB, including 5752 up-regulated genes and 3446 down-regulated genes, as well as 8555 DEGs in BT *vs* TC, including 5723 up-regulated genes and 2832 down-regulated genes. A total of 14,357 DEGs were found in TB *vs* BSB, including 7154 up-regulated genes and 7203 down-regulated genes, and 14,084 DEGs in TB *vs* TC, including 7188 up-regulated genes and 6896 down-regulated genes. The overall expression pattern of the four groups is shown in Fig. 2. Hierarchical cluster analysis indicated the similarity of genes or sample groups, consistent with the evaluation of biological replicates (Fig. 3). To determine the biological functions of DEGs between hybrid fish and parents, GO and KEGG pathway enrichment analyses were performed.

**Fig. 4:**
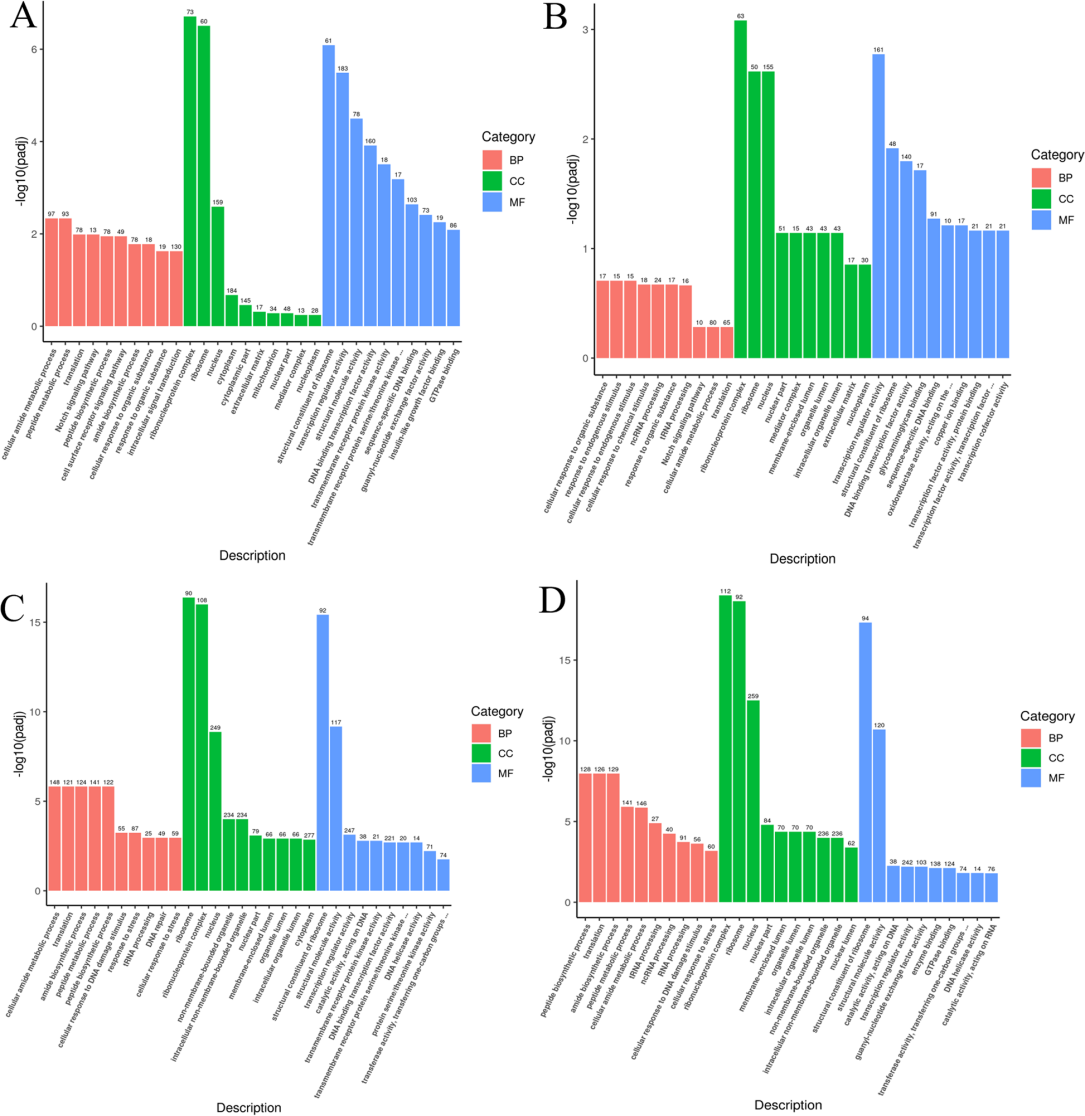
Gene Ontology (GO) functional classification of differentially expressed genes (DEGs) for four sample groups. **A **BT *vs* BSB, **B **BT *vs* TC, **C **TB *vs* BSB, **D **TB *vs* TC. The x-axis represents the GO term, and the y-axis shows the significance level of GO term enrichment (-log10(padj). Different colors indicate different functional classifications

**Fig. 5:**
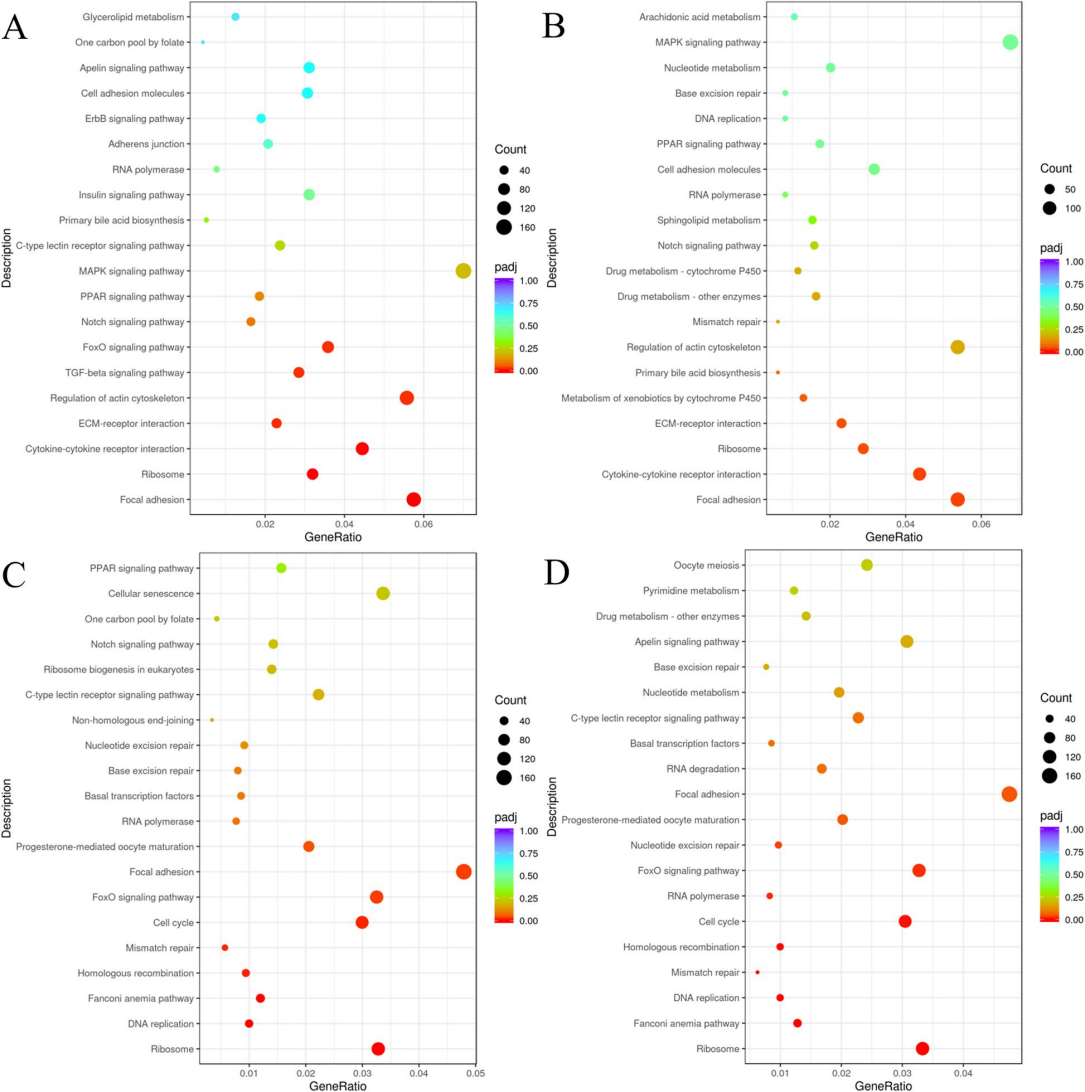
KEGG pathway enrichment analysis of differentially expressed genes (DEGs) for four sample groups: **A **BT *vs* BSB, **B **BT *vs* TC, **C **TB *vs* BSB, **D **TB *vs* TC. The x-axis represents the ratio of the number of differential genes annotated to the KEGG pathway to the total number of differential genes, and the y-axis represents the KEGG pathway. The size of the dots represents the number of genes annotated to the KEGG pathway, and the colors from red to purple represent the significance magnitude of enrichment

The GO analysis of DEGs in BT *vs* BSB showed enriched GO terms “cellular amide metabolic process” and “peptide metabolic process” in the biological process category, “ribonucleoprotein complex” and “ribosome” in the cell component category, and “structural constituent of ribosome” and “transcription regulator activity” in the molecular function category (*p* < 0.05) (Fig. 4A). KEGG enrichment analysis of DEGs in BT *vs* BSB revealed enriched pathways in “Focal adhesion” (dre04510), “Ribosome” (dre03010), “Cytokine-cytokine receptor interaction” (dre04060), “ECM-receptor interaction” (dre04512), “Regulation of actin cytoskeleton” (dre04810), “TGF-beta signaling pathway” (dre04350), and “FoxO signaling pathway” (dre04068) (*p* < 0.05) (Fig. 5A).

The GO analysis of DEGs in BT *vs* TC revealed enriched GO terms “ribonucleoprotein complex” and “ribosome” in the cell component category, “transcription regulator activity” and “structural constituent of ribosome” in the molecular function category, “cellular response to organic substance” and “response to endogenous stimulus” in the biological process category (*p* < 0.05) (Fig. 4B). KEGG enrichment analysis of DEGs in BT *vs* TC showed enriched pathways in “Focal adhesion” (dre04510), “Cytokine-cytokine receptor interaction” (dre04060), “Ribosome” (dre03010), and “ECM-receptor interaction” (dre04512) (*p* < 0.05) (Fig. 5B).

The GO analysis of DEGs in TB *vs* BSB revealed enriched GO terms “cellular amide metabolic process” and “translation” in the biological process category, “ribosome” and “ribonucleoprotein complex” in the cell component category, and “structural constituent of ribosome” and “structural molecule activity” in the molecular function category (*p* < 0.05) (Fig. 4C). KEGG enrichment analysis of DEGs in TB *vs* BSB showed significant enrichment pathways in “Ribosome” (dre03010), “DNA replication” (dre03030), “Fanconi anemia pathway” (dre03460) “Homologous recombination” (dre03440), “Mismatch repair” (dre03430), “Cell cycle” (dre04110), “FoxO signaling pathway” (dre04068), “Focal adhesion” (dre04510) and “Progesterone mediated oocyte maturation” (dre04914) (*p* < 0.05) (Fig. 5C).

The GO analysis of DEGs in TB *vs* TC showed enriched GO terms “peptide biosynthetic process” and “translation” in the biological process category, “ribonucleoprotein complex” and “ribosome” in the cell component category, and “structural constituent of ribosome” and “structural molecule activity” in the molecular function category (*p* < 0.05) (Fig. 4D). KEGG enrichment analysis of DEGs in TB *vs* TC showed enriched pathways in “Ribosome” (dre03010), “Fanconi anemia pathway” (dre03460), “DNA replication” (dre03030), “Mismatch repair” (dre03430), “Homologous recombination” (dre03440), “Cell cycle” (dre04110), “RNA polymerase” (dre03020), “FoxO signaling pathway” (dre04068), “Nucleotide excision repair” (dre03420), “Progesterone mediated oocyte maturation” (dre04914) and “Focal adhesion” (dre04510) (*p* < 0.05) (Fig. 5D).

### qRT-PCR validation of differentially expressed genes

To confirm the expression levels of differentially expressed genes identified by RNA-Seq in the gonads of hybrid fish and parents, 20 genes involved in biological events such as oocyte meiosis and homologous recombination were selected for qRT-PCR analysis. The expression profiles of these genes obtained from qRT-PCR were consistent with the RNA-Seq results (Additional file 2 Fig S1). The pearson correlation coefficient showed that the expression level was highly reliable (r = 0.94, *p* < 0.0001) for identifying significantly differentially expressed genes by RNA-seq ( Additional file 3 Fig S2).

### Enrichment analysis of differentially expressed genes after classification

The numbers of genes in the four expression patterns (ELD-B, ELD-T, OD and UD) in the two hybrid fish are shown in Table 2:

**Table 2:** Quantitative statistics of the four non-additive genes in BT and TB

Expression pattern	ELD-B	ELD-T	OD	UD
BT	1629	2121	4104	1083
TB	2124	2683	5732	4807

### Gene Ontology (GO) enrichment analysis of non-additive genes in hybrid fish

In BT, ELD-B genes were significantly enriched in transmembrane transporter activity, antiporter activity, and channel activity (Additional file 4 Fig. S3A). ELD-T genes were significantly enriched in metabolic process and protein dimerization activity (Additional file 4 Fig. S3B). OD genes were significantly enriched in signal transduction, cellular response to stimulus, transcription regulator activity, and enzyme binding (Additional file 4 Fig. S3C). UD genes were significantly enriched in cellular amide metabolic process, RNA processing, ribonucleoprotein complex, nucleus, and structural constituent of the ribosome (Additional file 4 Fig. S3D).

In TB, ELD-B genes were significantly enriched in transmembrane transporter activity, antiporter activity, and channel activity (Additional file 4 Fig. S4A). ELD-T genes were significantly enriched in metabolic process and protein dimerization activity (Additional file 4 Fig. S4B). OD genes were significantly enriched in signal transduction, cellular response to stimulus, ribonucleoprotein complex, transcription regulator activity and structural constituent of ribosome (Additional file 4 Fig. S4C). UD genes were significantly enriched in RNA processing, cellular response to DNA damage stimulus, nucleus, and catalytic activity (Additional file 4 Fig. S4D).

### KEGG enrichment analysis of non-additive genes in hybrid fish

**Fig. 6:**
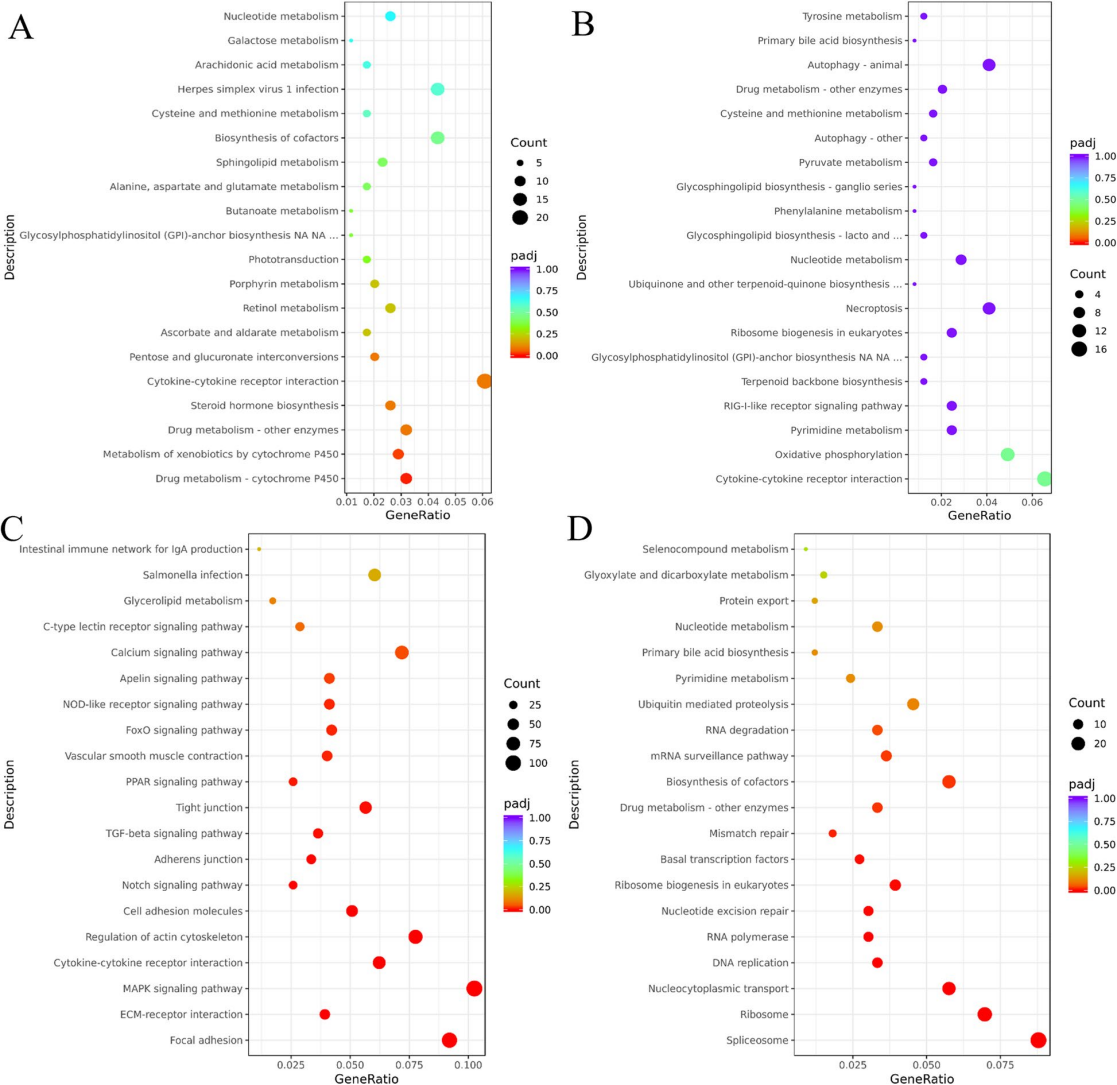
KEGG pathway enrichment analysis of non-additive genes in BT. **A **ELD-B, **B **ELD-T, **C **OD, **D**. UD

In BT, ELD-B genes were significantly enriched in Drug metabolism-cytochrome P450 and Metabolism of xenobiotics by cytochrome P450 pathways (*p* < 0.05). The Steroid hormone biosynthesis pathway was also among the top 20 enriched pathways (Fig. 6A). No pathways were significantly enriched in ELD-T genes, but among the top 20 pathways, the Cytokine-cytokine receptor interaction and Oxidative phosphorylation were ranked highest (Fig. 6B). OD genes were most enriched in Focal adhesion, ECM-receptor interaction and MAPK signaling pathway. Additionally, the TGF-beta signaling pathway and FoxO signaling pathway were also significantly enriched (*p* < 0.05) (Fig. 6C). UD genes were most enriched in Spliceosome and Ribosome pathways, with DNA replication, Nucleotide excision repair, and Mismatch repair also significantly enriched (*p* < 0.05) (Fig. 6D).

**Fig. 7:**
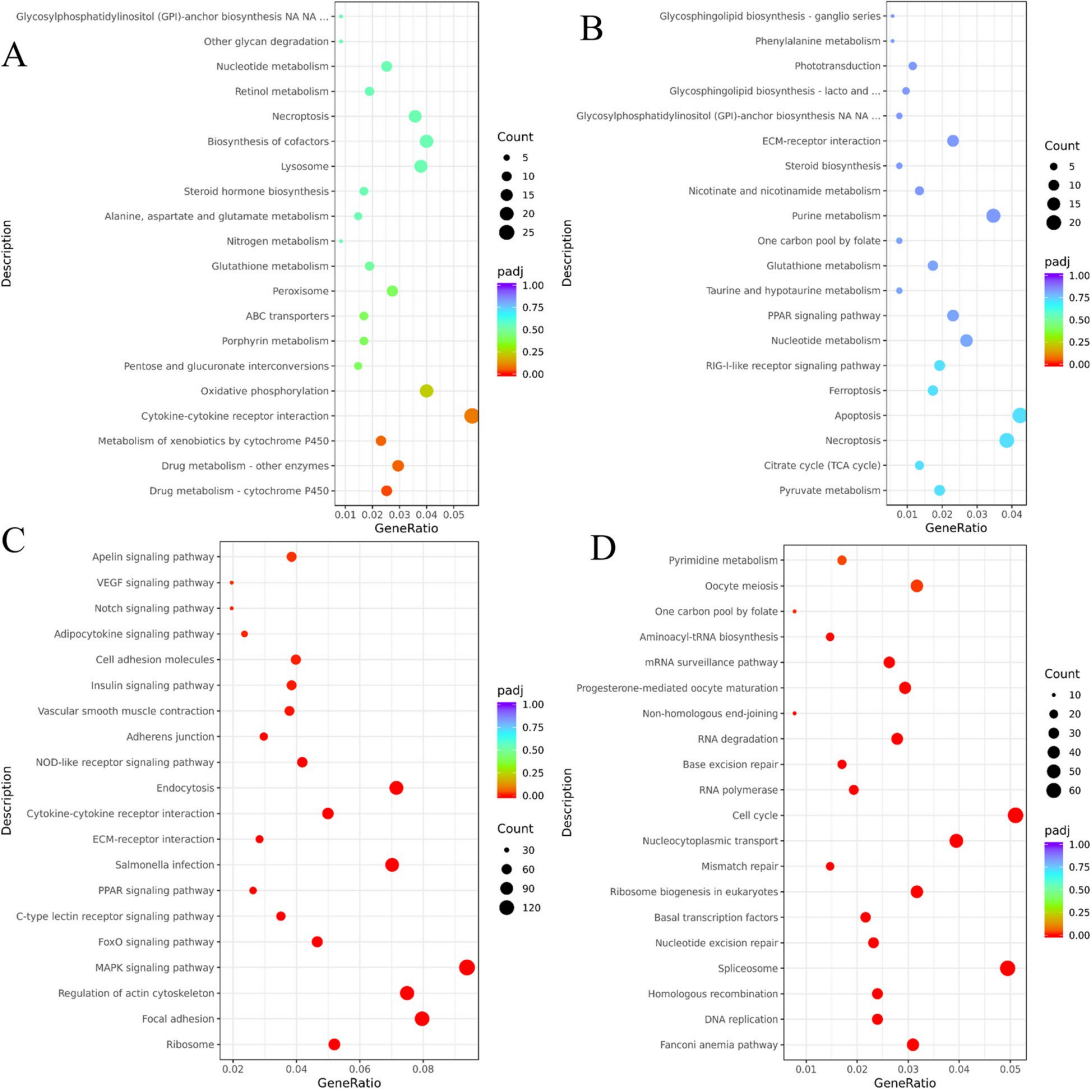
KEGG pathway enrichment analysis of non-additive genes in TB. **A **ELD-B, **B **ELD-T, **C **OD, **D **UD

In TB, ELD-B genes were significantly enriched in Drug metabolism-cytochrome P450 pathway (*p* < 0.05), with Metabolism of xenobiotics by cytochrome P450 and Steroid hormone biosynthesis pathways also among the top 20 enriched pathways (Fig. 7A). No pathways were significantly enriched in ELD-T genes, but among the top 20 pathways, the Pyruvate metabolism and Citrate cycle (TCA cycle) pathways were ranked highest (Fig. 7B). OD genes were most enriched in Ribosome, Focal adhesion and Regulation of actin cytoskeleton pathways. Additionally, the MAPK signaling pathway and FoxO signaling pathway were also significantly enriched (*p* < 0.05) (Fig. 7C). UD genes were most enriched in the Fanconi anemia pathway and DNA replication pathway, with Homologous recombination, Nucleotide excision repair, Mismatch repair, Cell cycle, Base excision repair, Non-homologous end-joining, Progestin-mediated oocyte maturation and Oocyte meiosis pathways also significantly enriched (*p* < 0.05) (Fig. 7D).

## Discussion

The BT and TB allodiploid hybrid fish, derived from artificial intergeneric hybridization between *M. amblycephala* and *C. alburnus*, are not only fertile in both sexes, but also form stable diploid lines with many heterosis traits. They represent a new fish germplasm resource with superior parental traits. According to previous research, when there are large differences in the number or karyotype of chromosomes between hybrid parents, inconsistent alleles from both parents in the hybrid can lead to gene regulation disorder, which can eventually lead to underdevelopment or even death of the individual [3]. Both *M. amblycephala* and *C. alburnus* have 48 chromosomes and the same karyotype [12], which may be an important reason for the fertility of BT and TB. Tang et al. [20] found that the heterochromosomes in the testes of BT-F_1_ can perform normal synapsis and segregation, and the gene sequences of *Dmc1* and *Rec8* are highly similar between *M. amblycephala* and *C. alburnus*. There was no abnormal gene expression in BT-F_1_, indicating that the main reason for the fertility of BT-F_1_ is that it can go through meiosis successfully. The allodiploid hybrid lineages of BT and TB provide a good material for studying the method of overcoming reproductive isolation in the hybrids of distant hybridization.

### Cytokines and transcription factors

There were 9198 DEGs in BT *vs* BSB, 8555 DEGs in BT *vs* TC, 14,357 DEGs in TB *vs* BSB, and 14,084 DEGs in TB *vs* TC. The number of DEGs between BT and parents was found to be less than that between TB and parents, indicating that there is less difference between BT and parents. No GO terms related to reproduction were enriched in the GO enrichment analysis results.

Focal adhesion plays vital roles in cell growth, death, cell cycle progression, cell proliferation, differentiation and repair [41, 42]. Cytokines are small molecular peptides that regulate cell functions and are essential in regulation of germ cell development, gametogenesis, gamete maturation [43], development of the testis and its normal function [44], physiological processes of the ovarian reserve, cell proliferation/differentiation folliculogenesis, and oocyte maturation [45]. In addition, cytokines create an immunologically permissive nutritional environment for embryonic development and are an important basis for reproductive success [45]. The transforming growth factor-B (TGF-beta) family is associated with testicular development and is involved in all stages of folliculogenesis [44, 45]. Members of the Foxo (forkhead protein O) transcription factor family are involved in several physiological processes in mammals, including metabolism, cell proliferation, apoptosis, and stress resistance [46]. Mammalian FoxO transcription factors include four members: FoxO1, FoxO3, FoxO4 and FoxO6, with FoxO1 and FoxO3 expressed in almost all tissues [46]. FoxO1 is required for self-renewal and differentiation of mouse spermatogonial stem cells as well as the initiation of spermatogenesis [47]. Liu et al. found that stem cell factor can inactivate the phosphorylation of FoxO3a, thereby inhibiting the apoptosis of rat oocytes, revealing that FoxO3a is involved in the regulation of oocyte apoptosis and primordial follicle formation in neonatal rat ovaries [48]. Extracellular matrix (ECM) is a macromolecule synthesized and secreted by cells, and it plays a crucial role in controlling cell survival, cell proliferation, cell differentiation and migration, and in supporting the establishment of organ structure during development [49, 50]. The differentially expressed genes between BT and TB and their parents were annotated in the above pathways, and these pathways were all up-regulated, suggesting that BT and TB promote gonad development and germ cell maturation through these pathways.

### Meiosis and DNA repair

During gonadal development, germ cells undergo mitotic proliferation and meiosis, during which biological events such as DNA replication, recombination, and exchange are highly active [51]. The FA (Fanconi anemia) pathway is involved in the cell cycle and homologous recombination, and together they play an essential role in gonadal sterility [52, 53]. Recent studies have shown that FA pathway is interconnected with homologous recombination, mismatch repair, and nucleotide excision repair pathways, which participate in germ cell proliferation, promote the meiotic process of germ cells, and maintain genome stability [51, 54]. However, hybridization of different species can destroy the genomic stability, and then affect the adaptability and fertility of hybrids [55]. Compared with the parents, the FA pathway, homologous recombination, mismatch repair, cell cycle and other pathways were all down-regulated in TB. It is hypothesized that the meiotic process of TB was inhibited. As the parents of TB are different species, subgenomic compatibility may be problematic, and the sites of homologous chromosome recombination during meiosis are reduced, resulting in the down-regulation of related pathways.

### Non-additive gene enrichment analysis

The MAPK (mitogen-activated protein kinase) signaling pathway is an important and highly conserved signal transduction pathways in animals, which can integrate different signals to regulate various cell functions. It plays a vital role in cell proliferation, differentiation, development, apoptosis and cell cycle regulation [56]. The MAPK cascade is involved in spermatogenesis, testicular development, and has important functions in sperm function, regulation of oocyte meiosis, spindle assembly, and ovulation [57–59]. Kisielnicka et al. found that the MAPK cascade promotes meiosis by coupling the degradation of RNA-binding protein at the stage of meiosis I in oocytes [60]. In *Caenorhabditis elegans*, OGR-2 inactivates MPK-1/ERK, a member of the MAPK pathway, by regulating the activity of lip-1 (MPK-1 phosphatase), thereby promoting the process of meiosis [61]. These findings indicate that the MAPK signaling pathway has important functions in animal reproduction. In BT and TB, OD genes were enriched in the MAPK signaling pathway, indicating that MAPK signaling pathway genes participate in cell cycle regulation, cell proliferation and differentiation of gonad, and may promote meiosis process, playing an essential role in the reproduction process of hybrid fish.

According to the studies [51–54], DNA repair pathways play a crucial role in promoting the process of meiosis and maintaining genome stability. In BT, UD genes were enriched in nucleotide excision repair and mismatch repair pathways, possibly because BT is a hybrid fish with two sub-genomes from different species, and its meiotic progression as well as genomic stability may be slightly affected. In TB, UD genes were enriched in Fanconi anemia pathway, homologous recombination, nucleotide excision repair, mismatch repair, cell cycle, base excision repair, non-homologous end joining, and oocyte meiosis pathway, probably because TB is similar to BT in possessing two sub-genomes, so that homologous chromosomes are not truly homologous, with meiotic recombination sites reduced, meiotic process inhibited, and genomic stability affected to a certain extent.

Cytochrome P450 hydroxylases catalyze the synthesis of various endogenous substances and the metabolism of exogenous compounds, such as steroid hormone synthesis and drug metabolism, respectively [62]. Steroid hormone biosynthesis in vertebrates mainly involves three conserved cytochrome P450 hydroxylases, namely CYP11A1, CYP17A1, and CYP19A1, which catalyze sequential steps of steroidogenesis [63]. Steroid hormones, which have a cyclopentane polyhydrophenanthrine structure, are bioactive compounds involved in various physiological processes in vertebrates and play crucial roles in reproductive development, such as regulating of cell proliferation and differentiation, developing sexual characteristics, and controlling the reproductive cycle [62, 63]. In BT and TB, the ELD-B genes were enriched in cytochrome P450 and steroid related pathways, indicating that the subgenome of *M. amblycephala* plays a dominant role in steroid hormone synthesis.

## Conclusion

In this study, we conducted transcriptome sequencing analysis to identify genes and pathways that are relevant to the reproductive characteristics of BT and TB. The results showed that differential gene expression between BT and parents did not significantly affect pathways directly related to meiosis, whereas the differential gene expressioin between TB and parents was enriched in pathways mostly related to meiosis, with these pathways down-regulated. This suggests that meiosis of BT may not be affected, but meiosis of TB may be suppressed.

Futhermore, we observed that there was no significant difference in the homologous recombination pathway between BT and parents, but this pathway was significantly downregulated in TB. These findings suggest that BT, a hybrid fish with *M. amblycephala* as maternal parent, has better genomic compatibility and fertility. Therefore, using *M. amblycephala* as maternal parent may be a viable approach for creating other hybrid fish strains with superior traits.

Our study helps to reveal the mechanism of crossing the reproductive barrier in hybrid fish, and has significant implications for understanding the reproductive characteristics of distant hybridization in fish.

### Supplementary Information

**Additional file 1:** **Tab. S1.** Primer sequence used in the real-time PCR.

**Additional file 2:** **Fig. S1.** Expression profiles of differentially expressed genes from RNA-Seq validated by qRT-PCR. A.Validation results for BT vs BSB and BT vs TC. B.Validation results for TB vs BSB and TB vs TC. The gene names and their full descriptions are provided below: sycp3: synaptonemal complex protein 3, hmgb1: high mobility group box 1, casp3b: caspase 3, apoptosis-related cysteine peptidase b, insra: insulin receptor a, bub3: BUB3 mitotic checkpoint protein, bcl2l11: BCL2 like 11, mlh3: mutL homolog 3, smc3: structural maintenance of chromosomes 3, tgfb1a: transforming growth factor beta 1a, igf1: insulin-like growth factor 1, rad51d: RAD51 paralog D, pcna: proliferating cell nuclear antigen, rpa3: replication protein A3, mapk12b: mitogen-activated protein kinase 12b, mapk11: mitogen-activated protein kinase 11, cdk1: cyclin dependent kinase 1, stag2a: stromal antigen 2a, samd1b: sterile alpha motif domain containing 1b, bcl2a: BCL2 apoptosis regulator a, gadd45ba: growth arrest and DNA-damage-inducible, beta a.

**Additional file 3:** **Fig. S2.** The pearson correlation coefficient between RNA-Seq and qRT-PCR.

**Additional file 4:** **Fig. S3.** GO enrichment analysis of non-additive genes in BT. (A)ELD-B (B)ELD-T (C)OD (D)UD. **Fig. S4** GO enrichment analysis of non-additive genes in TB. (A)ELD-B (B)ELD-T (C)OD (D)UD.

## Data Availability

The datasets generated during the current study are available from the corresponding authors upon reasonable request. The materials used in this study, including primers and plasmids, are available for non-commercial research purposes with a material transfer agreement (MTA), which can be obtained by contacting the corresponding authors. Any restrictions to the availability of data and materials will be disclosed upon request.

## Abbreviations

BSB: Blunt snout bream
TC: Topmouth culter
BT: The hybrid of *Megalobrama amblycephala* (♀) × *Culter alburnus* (♂)
TB: The hybrid of *Culter alburnus* (♀) × *Megalobrama amblycephala* (♂)
qRT-PCR: Realtime fluorescence quantitative PCR
DEGs: Differentially expressed genes
FPKM: Fragments Per Kilobase of exon model per Million mapped fragments
GO: Gene Ontology; KEGG: Kyoto Encylopaedia of Genes and Genomes
