# Supplementary material for: Comparative transcriptomic analysis of reproductive characteristics of reciprocal hybrid lineages derived from hybridization between Megalobrama amblycephala and Culter alburnus
Source: BMC Genom Data. 2023 Aug 12;24:45. doi: 10.1186/s12863-023-01141-6 (PMC10422732; doi:10.1186/s12863-023-01141-6)
Supplement: Supplementary file 2 — Additional file 2: Fig. S1. Expression profiles of differentially expressed genes from RNA-Seq validated by qRT-PCR. A.Validation results for BT vs BSB and BT vs TC. B.Validation results for TB vs BSB and TB vs TC. The gene names and their full descriptions are provided below: sycp3: synaptonemal complex protein 3, hmgb1: high mobility group box 1, casp3b: caspase 3, apoptosis-related cysteine peptidase b, insra: insulin receptor a, bub3: BUB3 mitotic checkpoint protein, bcl2l11: BCL2 like 11, mlh3: mutL homolog 3, smc3: structural maintenance of chromosomes 3, tgfb1a: transforming growth factor beta 1a, igf1: insulin-like growth factor 1, rad51d: RAD51 paralog D, pcna: proliferating cell nuclear antigen, rpa3: replication protein A3, mapk12b: mitogen-activated protein kinase 12b, mapk11: mitogen-activated protein kinase 11, cdk1: cyclin dependent kinase 1, stag2a: stromal antigen 2a, samd1b: sterile alpha motif domain containing 1b, bcl2a: BCL2 apoptosis regulator a, gadd45ba: growth arrest and DNA-damage-inducible, beta a. [file 12863_2023_1141_MOESM2_ESM.docx]

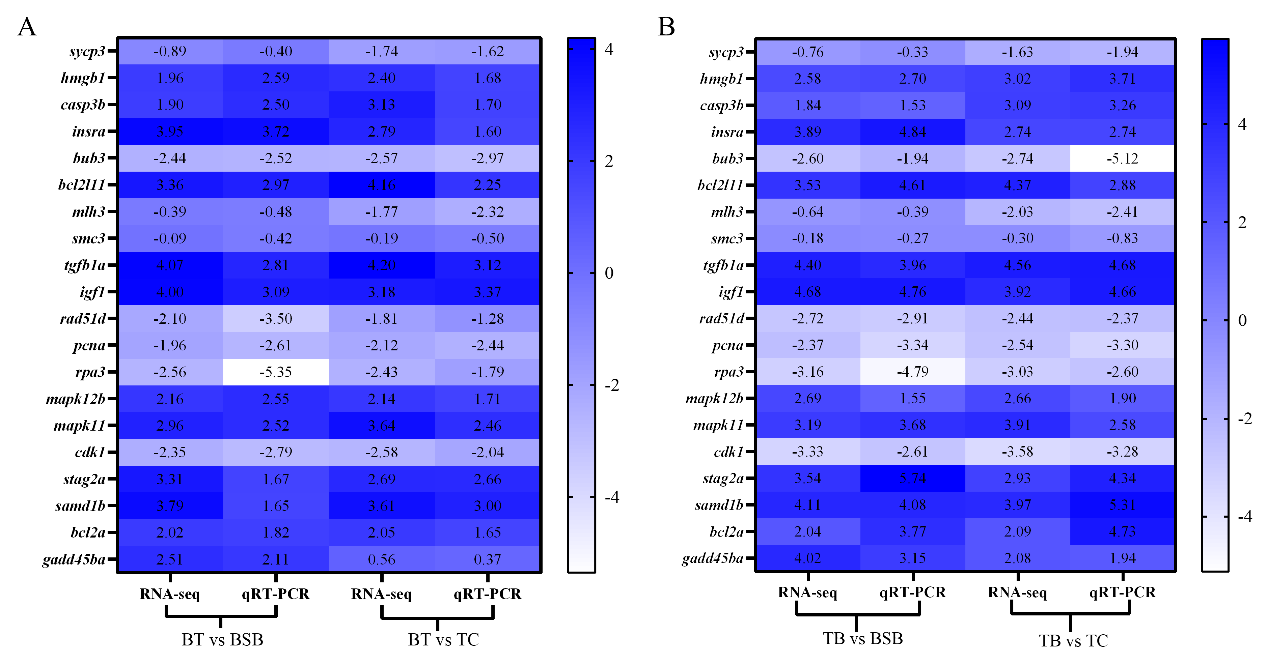
 Fig.S1 Expression profiles of differentially expressed genes from RNA-Seq validated by qRT-PCR. **A**.Validation results for BT vs BSB and BT vs TC. **B**.Validation results for TB vs BSB and TB vs TC. The gene names and their full descriptions are provided below:

*sycp3*: synaptonemal complex protein 3, *hmgb1*: high mobility group box 1, *casp3b*: caspase 3, apoptosis-related cysteine peptidase b, *insra*: insulin receptor a, *bub3*: BUB3 mitotic checkpoint protein, *bcl2l11*: BCL2 like 11, *mlh3*: mutL homolog 3, *smc3*: structural maintenance of chromosomes 3, *tgfb1a*: transforming growth factor beta 1a, *igf1*: insulin-like growth factor 1, *rad51d*: RAD51 paralog D, *pcna*: proliferating cell nuclear antigen, *rpa3*: replication protein A3, *mapk12b*: mitogen-activated protein kinase 12b, *mapk11*: mitogen-activated protein kinase 11, *cdk1*: cyclin dependent kinase 1, *stag2a*: stromal antigen 2a, *samd1b*: sterile alpha motif domain containing 1b, *bcl2a*: BCL2 apoptosis regulator a, *gadd45ba*: growth arrest and DNA-damage-inducible, beta a.
