# Supplementary material for: Comparative transcriptomic analysis of reproductive characteristics of reciprocal hybrid lineages derived from hybridization between Megalobrama amblycephala and Culter alburnus
Source: BMC Genom Data. 2023 Aug 12;24:45. doi: 10.1186/s12863-023-01141-6 (PMC10422732; doi:10.1186/s12863-023-01141-6)
Supplement: Supplementary file 3 — Additional file 3: Fig. S2. The pearson correlation coefficient between RNA-Seq and qRT-PCR. [file 12863_2023_1141_MOESM3_ESM.docx]

Fig. S2 The pearson correlation coefficient between RNA-Seq and qRT-PCR.
