# Supplementary material for: Comparative transcriptomic analysis of reproductive characteristics of reciprocal hybrid lineages derived from hybridization between Megalobrama amblycephala and Culter alburnus
Source: BMC Genom Data. 2023 Aug 12;24:45. doi: 10.1186/s12863-023-01141-6 (PMC10422732; doi:10.1186/s12863-023-01141-6)
Supplement: Supplementary file 4 — Additional file 4: Fig. S3. GO enrichment analysis of non-additive genes in BT. (A)ELD-B (B)ELD-T (C)OD (D)UD. Fig. S4 GO enrichment analysis of non-additive genes in TB. (A)ELD-B (B)ELD-T (C)OD (D)UD. [file 12863_2023_1141_MOESM4_ESM.docx]

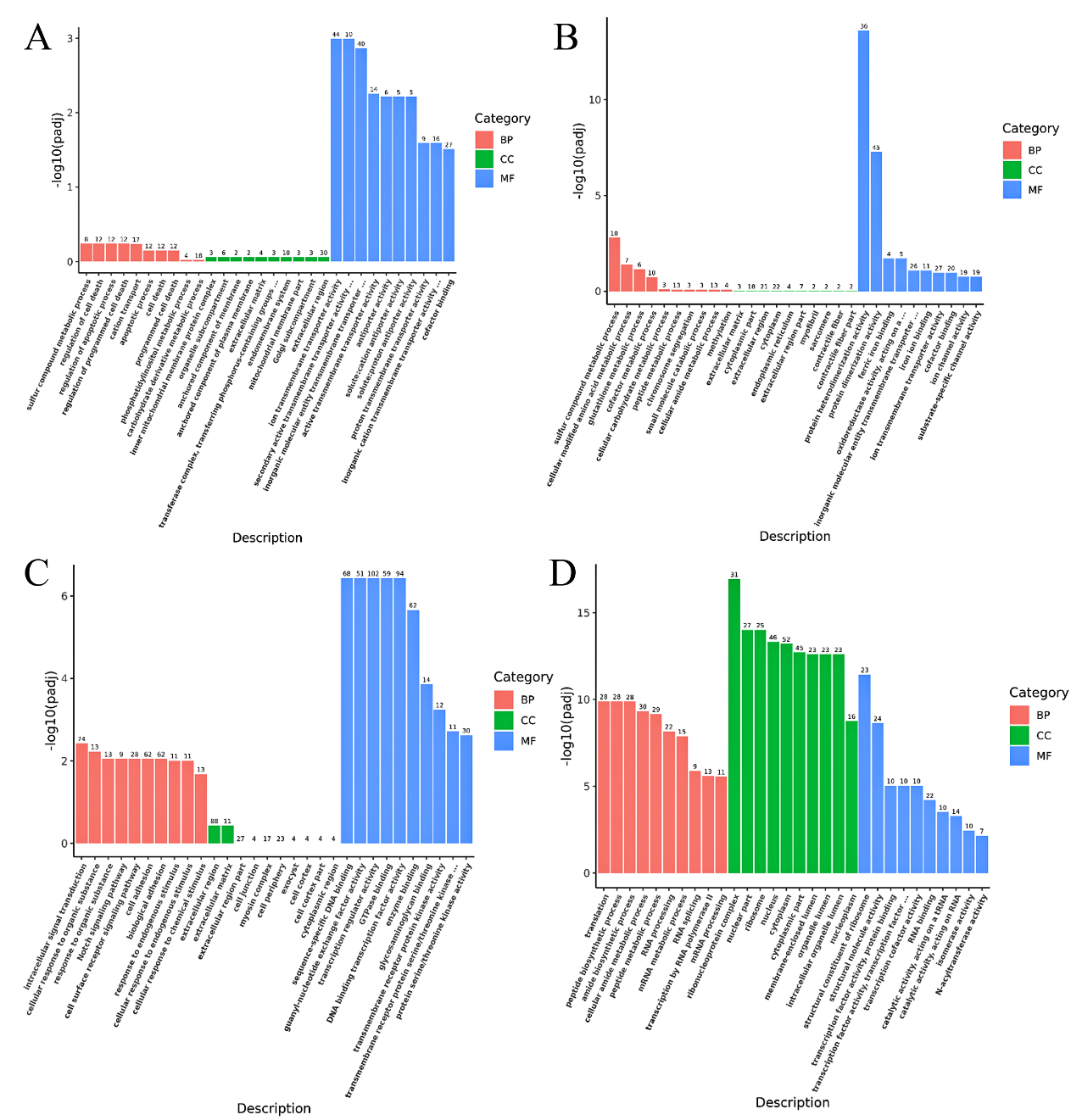


Fig. S3 GO enrichment analysis of non-additive genes in BT. (**A**)ELD-B (**B**)ELD-T (**C**)OD (**D**)UD.


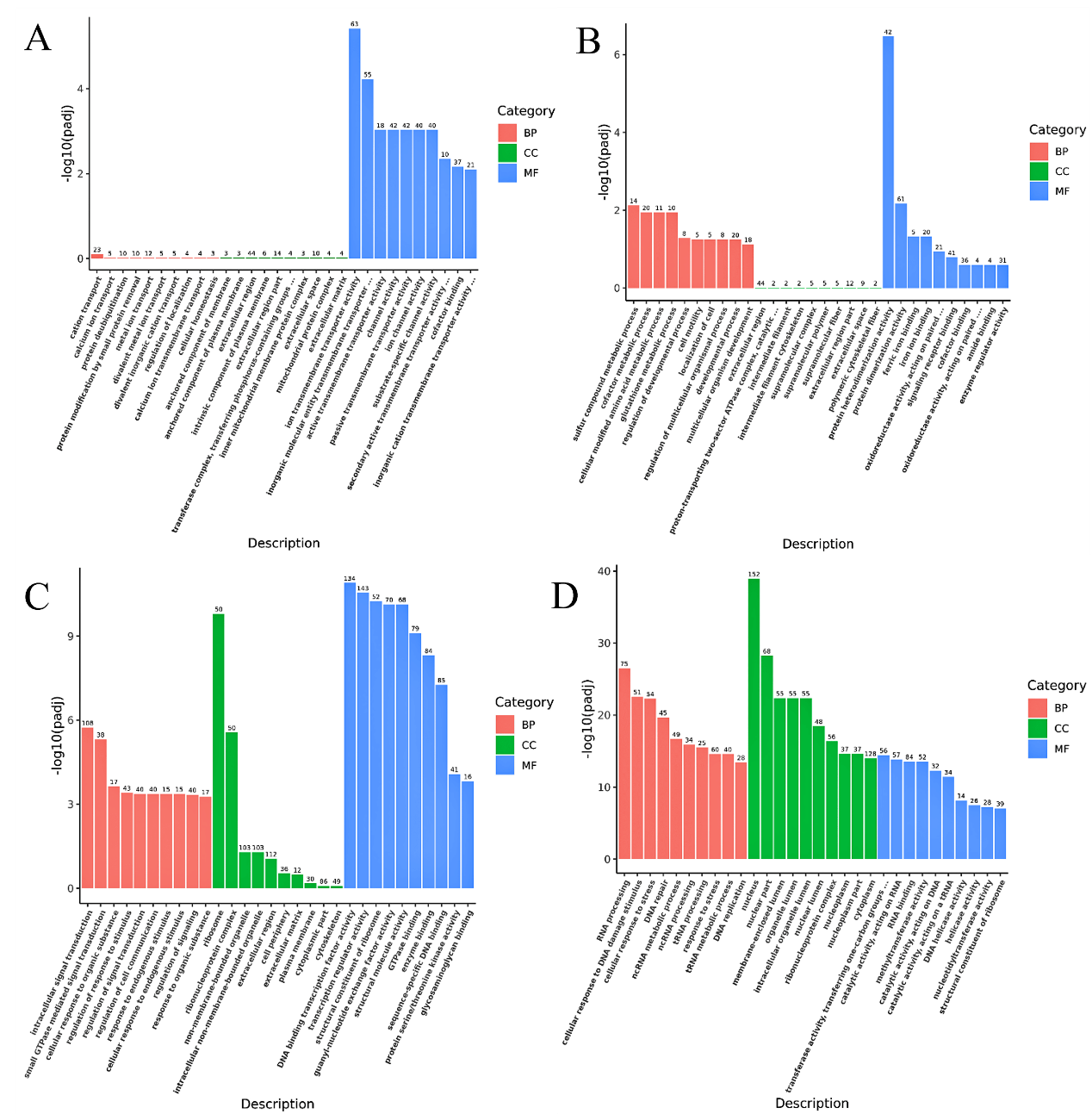


Fig. S4 GO enrichment analysis of non-additive genes in TB. (**A**)ELD-B (**B**)ELD-T (**C)**OD (**D**)UD.
